# Relationship between Body Mass Index (BMI) and testicular and hormonal parameters of sexually active male greater cane rats (*Thryonomys swinderianus*)

**DOI:** 10.21451/1984-3143-AR2019-0026

**Published:** 2020-02-05

**Authors:** Adenrele Olalekan Adebayo, Adebayo Kuyoom Akinloye, Bankole Olusiji Oke, Victor Olusegun Taiwo

**Affiliations:** 1 Department of Veterinary Anatomy, College of Veterinary Medicine, Federal University of Agriculture, Abeokuta, Nigeria; 2 Department of Veterinary Anatomy, Faculty of Veterinary Medicine, University of Ibadan, Ibadan, Nigeria; 3 Department of Veterinary Pathology, Faculty of Veterinary Medicine, University of Ibadan, Ibadan, Nigeria

**Keywords:** body mass index, hormones, obesity, orchidometry, greater cane rat

## Abstract

The current upsurge in intensive farming practices of greater cane rat has not only lead to higher growth rate but is accompanied by increased fat deposition especially in the males. This study attempts to characterize one of the most commonly used fat estimation parameter, the body mass index (BMI) as well as evaluates its relationship with testicular and hormonal parameters in seventy-two sexually active male cane rats over a period of one year. Six animals, kidded and raised in a farm, with known ages were used each month. The experimental protocols entail body measurements of weight, height and length; histology; orchidometry; and hormonal immunoassay of testosterone, estradiol, progesterone, LH and FSH using their various kits. The mean values of the body mass (BMI) and Lee (LI) indices of male greater cane rats were 1.18±0.20g/cm^2^ and 0.30±0.02g/cm respectively with the testicular histology indicating normal spermatogenesis. BMI/LI, both of which followed the same pattern, neither correlate with testicular parameters nor with serum testosterone, progesterone, LH and FSH concentrations but had low correlations with serum estradiol concentration (r^2^ = 0. 2; *p =* 0.0023). So, these relationships may provide clue on obesity and its effect on reproductive performance and strengthened the possibility of the characterized BMI/LI as obesity marker for breeding selection in male cane rat.

## Introduction

The greater cane rat (*Thryonomys swinderianus*), a wild hystricomorphic African rodent, is currently undergoing domestication and captive rearing in the West African sub region.This rat, popularly known as the Grasscutter, is highly exploited for its meat which is highly nutritive, relatively low fat content with greater percentage of lean meat (Opara, 2010). It is also regarded as the number one micro-livestock in the continent (Asibey and Addo, 2000). With the current drive for increase in the stock levels and the intensification of the production practices in cane rat farming (Adu et al., 2005), there is bound to be improved diet which will consequently lead to increased growth rate accompanied by a number of negative consequences, including an increase in fat deposition (Zerehdaran et al., 2004).

Obesity, the manifestation of excessive fat deposition in the body, is a condition caused by imbalanced energy homeostasis, and often associated with several morbidities such as type 2 diabetes, hypertension, coronary heart disease, testicular cancer, reduced fertility and osteoarthritis in both humans (Pasqualiet al., 2007; Papandreou et al., 2008) and rodents (Novelli et al., 2007). Although the effect of obesity on reproductive function has been well documented in literature (Sakamoto et al., 2008; Hammiche et al., 2012; MacDonald et al., 2013; Eisenberg et al., 2015) research into its impact on the reproductive health of males has been limited in comparison to the extensive research undertaken to investigate the female subfertility/infertility (Pasquali et al., 2007).Whilst there are strong evidences to suggest that obesity might affect male fertility, the variety of mechanisms through which it occurs have not been fully elucidated (ASRM, 2015). Therefore, a good understanding of the body mass index (BMI) and how it correlates with reproductive parameters will aid in furthering the knowledge of these mechanisms (MacDonald et al., 2013).

Body mass index (BMI) is an anthropometrical index commonly used to estimate body fat and define obesity in humans (Engeland et al., 2007), but can also be used for the same purposes in animals and birds (Pala et al., 2005; Mendeş et al., 2007). Reports on the relationship between BMI and reproductive parameters such as semen and testicular parameters are conflicting and confusing. For instance, Aggerholm et al. (2008) and MacDonald et al. (2013) reported no strong relationship between BMI and sperm concentration or total sperm count in humans, but Qin et al. (2007) and Hammiche et al. (2012) documented strong positive correlation between these same parameters. While, Bahk et al. (2010) reported that in humans, there remains some controversy regarding whether testicular volume is related to BMI, there is complete lack of information on the BMI and its relationship with either testicular parameters or semen parameters in the greater cane rat.

Therefore, this work attempts to characterize BMI and evaluate its correlations with testicular parameters - volume, weight, length and diameter in a population of sexually active male greater cane rat raised under intensive management system. This is to provide baseline/preliminary information that will be useful in the definition and study of obesity as well as the mechanism of its adverse effects on reproductive functions in this rat.

## Methods

### Animal management

This study was carried out for twelve (12) calendar months at the grasscutter domestication facility of the Federal University of Agriculture, Abeokuta, Nigeria. The experimental protocols followed the ethical guidelines and approval (ethical code no: ethics 03/14/04) of the Animal care Committee of the Federal University of Agriculture, Abeokuta, Nigeria. All procedures and handling of the rats also followed the guide for the care and use of experimental animals (National Institute of Health (NIH), USA. Seventy two (72) sexually matured (age range: 7-24months and weight range: 1420-3040g), adult male greater cane rats, kidded and raised in the grasscutter Farm, with known reproductive and medical records, were used. Six (6) males were used each month for the twelve months of the year. All the animals had brownish perineal staining which is usually used as index of sexual maturity in male cane rat (Adu and Yeboah, 2003). They were maintained on commercial cane rat feed and Elephant grass stems with water given *ad libitum*.

### Body measurements and Estimation of BMI

After light inhalation anaethesia, the weight, height and nose-to-anus length of each cane rat was taken. Weights were taken using the Mettler’s weighing balance; heights were measured from the scapular point to the ankle while the lengths were measured from the tip of the nose to the anus. These parameters were recorded against the known age of each animal. The body mass and Lee indices for each animal were calculated as recommended by Novelli et al. (2007):

BMI = body weight (g) divided by the square of the nose-to-anus length (cm).

*Lee Index = cube root of body weight (g) divided by nose-to-anus length (cm).

*The Lee index was added to further confirm and verify the result of the BMI.

### Blood sampling and testicular tissue measurements

Blood samples were collected twice per day for seven times within the month, from each animal after which its transcardially perfused with Karnovsky’s fixative and opened-up through a mid-ventral abdominal incision. The ischiatic arch was completely disarticulated to expose the reproductive organs and the testes were carefully dissected out individually. Testicular weight, length, width and diameter were measured for each animal using microwa analytical balance and vernier caliper while the testicular volume was estimated by water displacement method. Serum samples were separated from the collected blood for hormonal immunoassay and testicular tissue samples were taken for histology.

### Histology procedure

Testicular samples for the histology were further fixed in Karnovsky’s fixative, dehydrated in graded series of ethanol, cleared in xylene and paraffin-embedded. Five-micrometre-thick sections were cut and mounted on gelatinized slides, stained with H&E and examined with Axioskop 2 plus, Carl Zeiss light microscope (Germany).

### Hormonal immunoassay

The serum levels for testosterone, estrogen, progesterone, luteinizing (LH) and follicle stimulating (FSH) hormones were assayed for each of the six animals in each month of the year using the Microplate Immunoenzymometric assay kits specific for each hormone. For testosterone, the DS-EIA-STERIOD-TESTOSTERONE-RT kit (Interco Diagnostic Ltd, UK) was used while for estrogen, ESTRADIOL-ELISA test kit (Fortress Diagnostics Ltd, UK) was employed. The progesterone kit used was the DS-EIA-STEROID-PROGESTERONE-RT (Interco Diagnostic Ltd, UK) while DS-EIA-GONADOTROPIN-LH (Interco Diagnostic Ltd, UK) and DS-EIA-GONADOTROPIN-FSH (Interco Diagnostic Ltd, UK) were used for the luteinizing and follicle stimulating hormones respectively. The test procedure according to user instruction for each kit was duly followed. Briefly, 25µl of each of the Calibrators (serum reference for the hormone at graded concentrations), control serum and sample serum of each cane rats were pipetted into appropriately labeled Anti-hormone-coated microtiter wells in duplicate. 10µl of the Conjugate (monoclonal anti-hormone-antibodies conjugated with horse radish peroxidase) was added to each well, swirled for 20-30 seconds to mix, covered and incubated for 60minutes at room temperature. The content of the microtitre were then decanted and blot-dried with absorbent tissue paper. To each well, 300µl of reconstituted washing solution (prepared by mixing the concentrated Washing Solution and distilled water at ratio 1:25 in a separate jar) was added, decanted and blot-dried. This washing was repeated four (4) additional times, after which 100µl of TMB-Substrate was pipetted into each well at timed intervals and incubated for 15-20minutes at room temperature in a dark cupboard. The reaction was then stopped by the addition of 150µl of the Stopping reagent (0.2M sulphuric acid solution) into each well at timed intervals and the microtitre wells read on an ELISA reader (Elx 800, BioTek, England).

The serum concentration of the hormone in each sample was estimated on a 4-parameter calibrator curve plotted with the optic densities/Absorbance on the Y-axis and calibrator concentration on the X-axis. All the test validation criteria for each of the assay were met in this work in accordance with the kit manufactures’ instructions. After standardization the intra-assay and inter-assay coefficients (% CV) were calculated from the duplicates and the multiple assay plates respectively. These were then compared with that of the kit manufacturer’s calculated CV which is less than 6% CV for intra-assay and 9% for inter-assay.

### Statistical analysis

Data were expressed as mean ± standard error. Pearson’s correlation analysis was used to examine the relationship within and between data using Paleontological Statistics version 2.15 (PAST) data analysis tool. *P*-value ˂ 0.05 was considered statistically significant.

## Result

**Figure 1:**
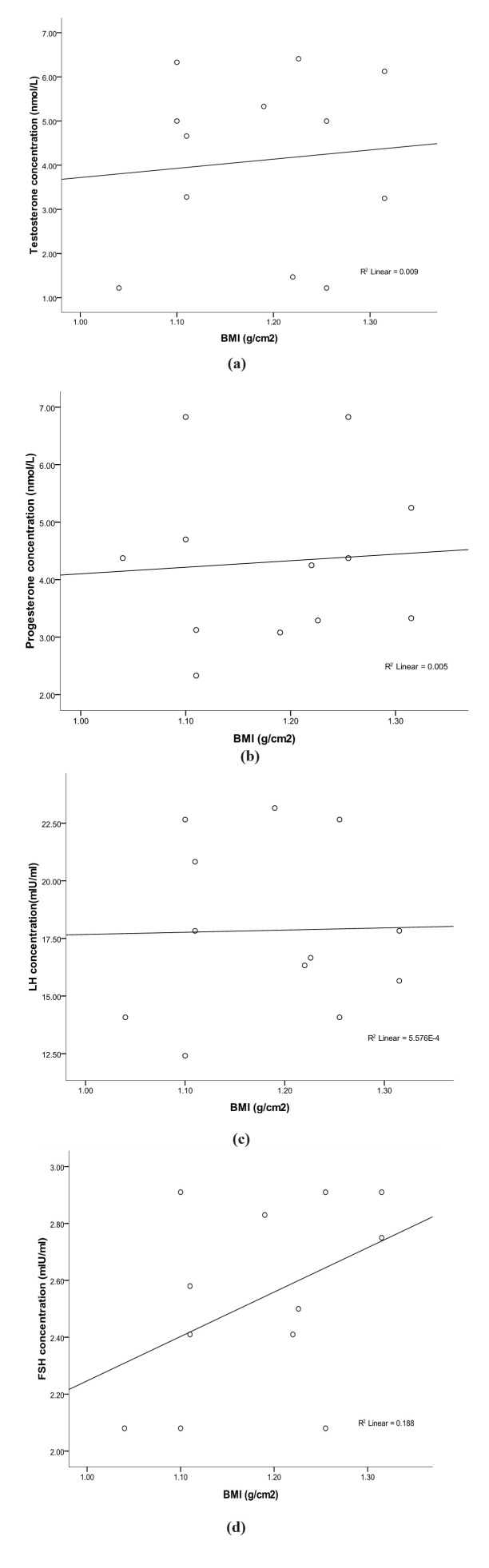
(a-d) Scatter plots showing the relationship between the body mass index and the serum (a) Testosterone, (b) Progesterone (c) LH and (d) FSH concentrations in the greater cane rat. Each plot represents the mean of six samples and shows the linear correlation co-efficient (r^2^).

**Figure 2:**
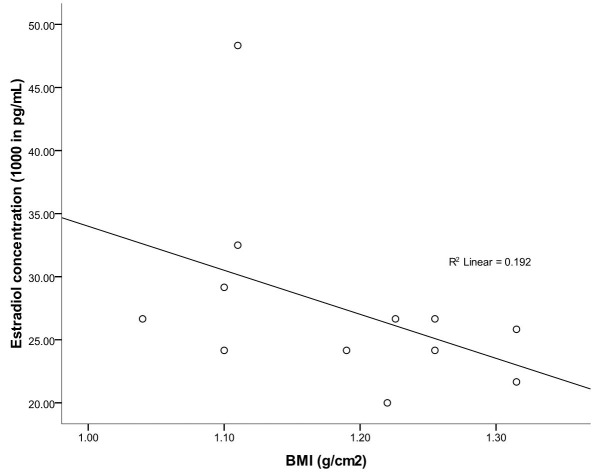
Scatter plot of the correlation between the body mass index and the serum estradiol concentration in the greater cane rat. Each plot represents the mean of six samples and shows the linear correlation co-efficient (r^2^).

**Figure 3:**
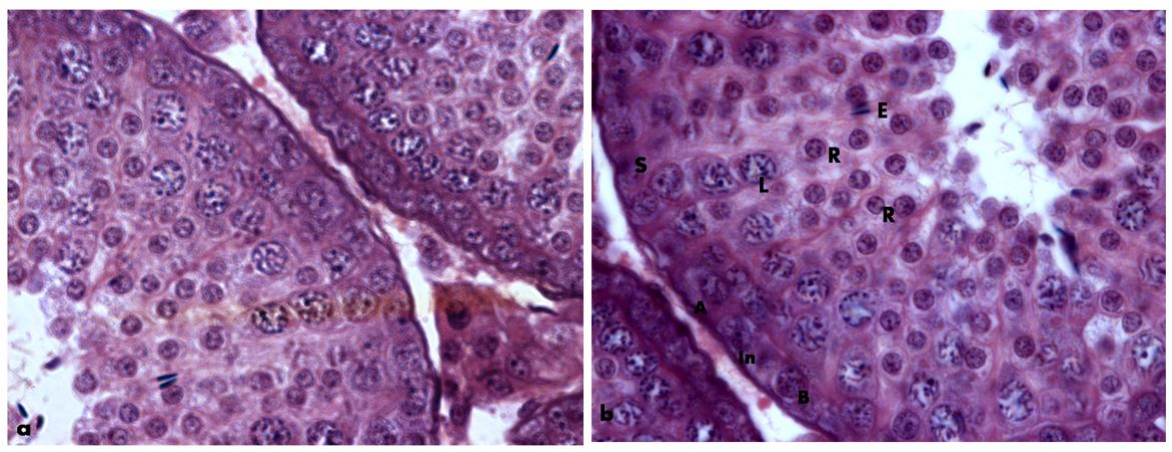
Normal histo-architecture of the testes showing the spermatogenic cells; Spermatogonia (A, B, In), spermatocytes (L) and spermatids (R, E); Sertoli (S) cells in the greater cane rat. H&E. Scale bar: 25µm.

The mean values of the body mass (BMI) and Lee (LI) indices of male greater cane rats, at age range of 7-24months and body weight range of 1.42-3.04kg, were observed to be 1.18 ±0.20g/cm^2^ and 0.30 ±0.02g/cm respectively (Table 1). While there was a significantly positive relationship between the animal height and the testicular weight and volume (r^2^= 0.44 and r^2^ = 0.21 respectively), no correlation was observed between length of the animals and the testicular weight and volume. In the same vein, neither the BMI nor LI showed any correlation with the testicular weight and volume in the greater cane rat (Table2). The scatter-grams showed the same pattern for both BMI and LI, having no correlation with serum testosterone, progesterone, LH and FSH concentrations (Figure 1ad) but low correlations with serum estradiol concentration (r^2^ = 0. 2) (Figure 2). The histology of the testes showed normal histo-architecture indicative of typical spermatogenesis (Figure 3).

**Table 1:** The mean, standard deviation and range of Age, body parameters, gross testicular morphometric data and anthropometric values in the male greater cane rat.

	**Mean**	**±SD**	**Range**
*Age (Months)*	12.8	±6.15	7-24
*Body weight (kg)*	2.23	±0.40	1.42-3.04
*Body length (cm)*	43.6	±3.17	37-50.5
*Height (cm)*	16.36	±1.04	14.5-19
*Testicular weight (g)*	1.43	±0.40	0.84-2.57
*Testicular volume (cm^3^)*	1.33	±0.26	1-2
*Testicular diameter (cm)*	1.10	±0.13	0.9-1.5
*BMI (g/cm^2^)*	1.18	±0.20	0.88-1.70
*Lee index (g/cm)*	0.30	±0.02	0.27-0.35

**Table 2:** Correlation co-efficients between age, body measurements, testicular morphometric and anthropometric parameters in the male greater cane rat.

	**Age (months)**	**Body weight (g)**	**Body length (cm)**	**Height (cm)**	**Testicular weight (g)**	**Testicular Volume (cm^3^)**		**BMI (g/cm^2^)**	**Lee Index (g/cm)**
*Age (months)*	1								
*Body weight (g)*	0.57	1							
*Body length (cm)*	0.34	0.56	1						
*Height (cm)*	-0.09	0.33	0.17	1					
*Testicular weight (g)*	-0.18	0.01	-0.04	0.44*	1				
*Testicular Volume (cm^3^)*	-0.13	0.09	0.03	0.21**	0.68	1			
*BMI (g/cm^2^)*	0.34	0.61	-0.25	0.01	-0.04	0.01	1		
*Lee index (g/cm)*	0.09	0.27	-0.64	0.10	0.02	0.01	0.84		1

* p= 0.0012;

** p=0.0043.

## Discussion

This study reports estimated mean values for the body mass (BMI) and Lee (LI) indices for a population of sexually active greater cane rat. While the incidence of obesity is on the increase especially among pet animals (German, 2006), most information on BMI and other fat estimation parameters on animals are generally few. With the current drive in the domestication of the greater cane rat, these BMI and LI estimates can serve as standard obesity markers and aid in selecting males for breeding.

Age and puberty are important factors in the determination of normal BMI and LI in sexually matured males (Novelli et al., 2007; Lu et al., 2015). In the rat, these indices were estimated based on the age of cessation of growth and development as well as the age from when the mean BMI values became constant (Novelli et al., 2007). Although the age of cessation of growth and development was yet to be determined in the greater cane rat, it was observed from this work that at sexual maturity, there was no significant relationship between ages of these animals and these indices. So, it can be inferred that the estimated BMI and LI in this work represent the normal values in sexually matured male greater cane rat. These observed values were also comparable with the BMI and LI values in the Wistar rat (Novelli et al., 2007).

Testicular weight and volume are good indicators of normal spermatogenesis particularly when compared with typical histo-architecture of the testes (Bahk et al., 2010). They can also correlate with both sperm quality and quantity (Sakamoto et al., 2008; Bahk et al., 2010). In the greater cane rat, testicular volume and weight correlate with the height but not the body weight or length of the animal. In humans, whereas close relationship between orchidometric parameters and height has been reported (Handelsman and Staraj, 1985; Bahk et al., 2010), their relationship with body weight is controversial. Wikramanayake (1995) and Spyropoulos et al. (2002) reported relationship between testicular volume and body weight while Sobowale and Akiwumi (1989) and Bahk et al. (2010) reported no correlation. The recent account of Innocent et al. (2016) however reported relationship between left testicular volume and body weight but no relationship between right testicular volume and body weight. The observation in the cane rat reported in this work is consistent with our previous findings (Adebayo et al., 2009).

In this study there was no correlation between either BMI or LI and testicular weight or volume. This was similar to what had been reported in young sexually active human adults (Bahk et al., 2010; Innocent et al., 2016). In the same vein, the observed lack of relationship between BMI and the serum levels of testosterone, progesterone, LH and FSH as well as the low correlation between BMI and the serum estradiol level in the greater cane rat was consistent with what obtained in man (Aggerholm et al., 2008; MacDonald et al., 2013). In man, increased adipose tissue which showed as higher BMI is associated with changes in the male reproductive hormone profile causing alterations in the levels of testosterone and estrogen as well as sex-hormone binding globulin (SHBG) (Pasquali et al., 2007; ASRM, 2015). These hormonal abnormalities were said to be due to increased peripheral conversion of androgen to estrogen associated with the increased adipose tissue present at a higher BMI (Pasquali et al., 2007; ASRM, 2015). In the light of the above observations in humans, the relationship between the BMI and the serum estradiol seen in the cane rat is plausible giving the lack of correlation between these indices and serum testosterone level. It can therefore be inferred that higher BMI beyond this estimated values can alter hormonal balance which might affect reproductive performance in the male cane rat.

## Conclusion

This work has shown that, the relationship between BMI/LI (in young sexually active greater cane rat) and both the testicular parameters and reproductive hormones, seems to have similar pattern to that reported in young sexually active humans. The correlation of these indices with the serum estradiol may provide a possible clue to the mechanism by which obesity in this animal can affect its reproductive performance, although further works are still needed. All these have strengthened the possibility of these characterized BMI/LI as a standard obesity marker for breeding selection of the male greater cane rat.
